# Clinicopathological Features of Esophageal Carcinosarcoma Treated with Surgery: A Series of Nine Cases

**DOI:** 10.70352/scrj.cr.26-0080

**Published:** 2026-07-04

**Authors:** Kohei Saisho, Satoru Matono, Naoki Mori, Yutaro Mihara, Masashi Nakagawa, Hideaki Kaku, Yuya Tanaka, Yuichi Goto, Takefumi Yoshida, Taro Isobe, Tomoya Sudo, Hisamune Sakai, Nobuya Ishibashi, Toru Hisaka, Jun Akiba, Fumihiko Fujita

**Affiliations:** 1 Department of Surgery, Kurume University School of Medicine, Kurume, Fukuoka, Japan; 2 Department of Pathology, Kurume University School of Medicine, Kurume, Fukuoka, Japan

**Keywords:** esophageal carcinosarcoma, esophagectomy, lymphovascular invasion, lymph node metastasis

## Abstract

**INTRODUCTION:**

Esophageal carcinosarcoma (ECS) is a rare malignant tumor composed of both carcinomatous and sarcomatous components. Owing to its rarity, the clinicopathological characteristics, optimal treatment strategies, and prognostic factors of ECS remain poorly understood. This study reported the clinicopathological features and treatment outcomes of surgically treated ECS.

**CASE PRESENTATION:**

Between 1999 and 2019, 713 patients underwent surgery for thoracic esophageal malignancies at Kurume University Hospital. Among them, 9 patients (1.3%) were diagnosed with ECS. All patients were male, with a median age of 65 years (range, 58–76 years). Preoperative endoscopic biopsy correctly diagnosed ECS in only 1 patient, while the majority were initially diagnosed as squamous cell carcinoma. Recurrence occurred in 5 patients, including local recurrence, lymph node metastasis, and distant metastasis. The 5-year overall survival rate was 22.2%, reflecting a generally poor prognosis. Histopathological examination revealed that the histological component identified at sites of lymphatic invasion in the primary tumor was consistent with those observed in metastatic lymph nodes.

**CONCLUSIONS:**

ECS may exhibit aggressive biological behavior that is not adequately reflected by tumor depth alone. Surgery alone may be insufficient for disease control in some patients. Further studies are required to clarify the biological characteristics of ECS and to establish optimal treatment strategies.

## Abbreviations

DCF
: docetaxel, cisplatin, and 5-fluorouracil
DSS
: disease-specific survival
ECS
: esophageal carcinosarcoma
SCC
: squamous cell carcinoma

## INTRODUCTION

**Fig. 1:**
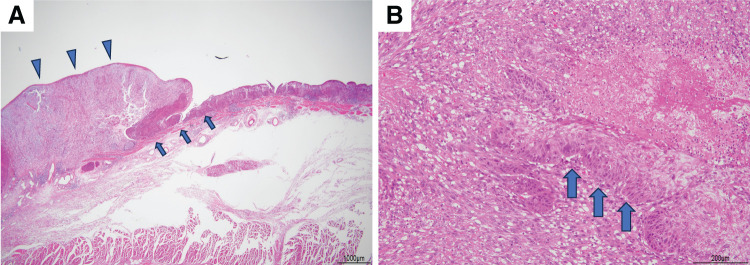
(**A**) Loupe appearance of the tumor. The polypoid lesion consisted of a sarcomatous component (arrowheads), and at the margin, a carcinomatous component (arrows). (**B**) Magnified view. The carcinomatous component (arrows) was surrounded by sarcomatous cells.

ECS is a rare malignant neoplasm characterized by both carcinomatous and sarcomatous components (**Fig. 1A** and **1B**).^1)^ It accounts for 0.2%–1% of all esophageal malignancies.^2–8)^ Although surgical resection is currently the primary treatment modality for ECS, a definitive treatment protocol has not been established.^9)^ Consequently, the prognosis remains poor, with a 5-year survival rate of 26%–48%.^3–5)^ Accumulating more clinical cases is essential to identifying optimal treatment strategies.

In this study, we report the clinicopathological characteristics and treatment outcomes of 9 patients who underwent surgery for ECS.

## CASE PRESENTATION

Between January 1999 and December 2019, 713 patients with primary thoracic esophageal malignant tumors underwent esophagectomy at Kurume University Hospital. Among them, 9 patients (1.3%) diagnosed with ECS were included in this study. We analyzed the clinicopathological features of these 9 patients; the median duration of follow-up after esophagectomy was 22 months (range: 5–139 months). The clinicopathological diagnosis and staging were classified according to the Union for International Cancer Control (UICC) TNM classification, 8th edition. Lymph node stations were described according to the Japanese Classification of Esophageal Cancer, 12th edition. Clinical response to chemotherapy or to chemoradiotherapy was evaluated using the Response Evaluation Criteria in Solid Tumors (RECIST) version 1. Survival analysis was performed using the Kaplan–Meier method with JMP software (version 16; SAS Institute, Cary, NC, USA).

Notably, we examined the pathological findings of lymph node metastases in detail. In patients with lymphatic invasion, the concordance between the histology at the site of the lymphatic invasion and that of the metastatic lymph node was investigated. The presence or absence of sarcomatous components was determined based on morphological findings and on immunohistochemical staining for vimentin using the anti-vimentin antibody (clone V9, Dako; Agilent Technologies, Santa Clara, CA, USA; this antibody is now discontinued). Immunohistochemical staining was performed using the Ventana Benchmark system (Ventana Medical Systems, Tucson, AZ, USA).

Written informed consent was obtained from each patient. The study protocol adhered to the ethical guidelines of the Declaration of Helsinki and its later amendments and was approved by the Ethics Committee of Kurume University (Approval Number: 2025-144).

## RESULTS

The clinicopathological features are summarized in **Table 1**. All patients were male, with a median age of 65 years (range: 58–76 years). The most common tumor location was the middle thoracic esophagus (6 cases), followed by the lower and upper thoracic esophagus. The median tumor size was 10 cm (range: 4–18 cm), indicating a predominance of large tumors. Regarding macroscopic type, Type 1 or Type 1 + 0-IIc predominated, accounting for 7 cases (78%). Preoperative diagnosis by endoscopic biopsy was SCC in most cases, while ECS was diagnosed in only 1 case (11%).

**Table 1:** Clinicopathological characteristics of the 9 patients with esophageal carcinosarcoma

Case no.	Age (years)	Gender	Tumor location	Tumor size (cm)	Tumor type	Biopsy findings	cT	cN	cStage	pT	pN	pStage	Ly	V	Component of lymphatic invasion site	Component of lymph node metastasis	Adjuvant chemotherapy	Recurrence	Cause of death	Survival time (months)
1	73	Male	Mt	11	1	Carcinoma	3	3	III	2	3	III	+	+	NA	Carcinomatous component	−	−	Other disease	15
2	63	Male	Lt	10	1 + 0-IIc	SCC	3	0	II	1b	0	I	+	+	NA		−	−	Other disease	139
3	58	Male	Mt	4	1	SCC	3	2	III	1b	0	I	+	+	NA		−	+	Esophageal carcinosarcoma	22
4	60	Male	Mt	4	1 + 0-IIc	SCC	2	1	II	2	2	III	+	+	Carcinomatous component	Carcinomatous component	+	+	Esophageal carcinosarcoma	35
5	50	Male	Mt	11	1	Sarcomatous carcinoma	3	2	III	2	1	II	+	+	Sarcomatous component	Sarcomatous component	+	+	Esophageal carcinosarcoma	5
6	65	Male	Lt	5	0-Ip + 0-IIc	SCC	1b	0	I	1b	1	II	+	+	Carcinomatous component	Carcinomatous component	+	+	Gallbladder cancer	22
7	65	Male	Mt	18	1	Pleomorphic sarcoma	4a	0	III	1b	0	I	−	−			−	−	Lung cancer	112
8	72	Male	Ut	4	2 + 0-IIc	SCC	2	1	II	1b	0	I	−	−			−	−	Other disease	20
9	76	Male	Mt	10	1 + 0-IIc	SCC	3	2	III	1b	0	I	−	+			−	+	Esophageal carcinosarcoma	26

Sites of lymph node metastasis (cases 1, 4, 5, and 6): 105, 106recR, 106recL, 107, 110, 112aoA, 1, 2, 3, and 7.

Lt, lower thoracic esophagus; Ly, lymphatic invasion; Mt, middle thoracic esophagus; NA, not available; SCC, squamous cell carcinoma; Ut, upper thoracic esophagus; V, venous invasion

The operative procedure consisted of esophagectomy via a right-thoracoabdominal approach followed by esophageal reconstruction using a gastric tube in 8 cases (89%). One patient underwent distal esophageal resection via a left thoracoabdominal approach. Four patients presented with lymph node metastases, and 3 of whom received adjuvant chemotherapy with cisplatin and 5-fluorouracil.

**Fig. 2:**
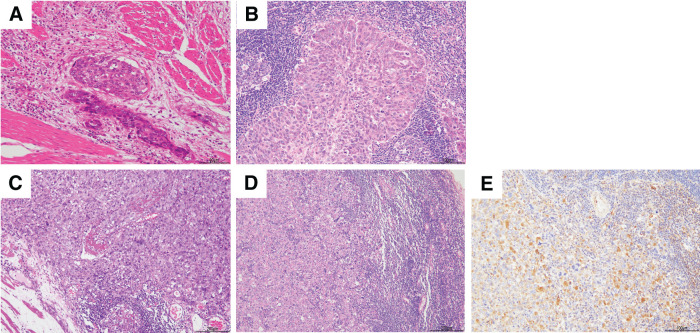
Histopathological findings of representative cases showing lymphovascular invasion in the primary tumor and corresponding lymph node metastasis (Cases 4 and 5). (**A**) Hematoxylin–eosin staining of the lymphatic invasion site in Case 4. A carcinomatous component was observed in the lymphatic invasion site. (**B**) Hematoxylin–eosin staining of the metastatic lymph nodes in Case 4. A carcinomatous component was observed in the metastatic foci. (**C**) Hematoxylin–eosin staining of the lymphatic invasion site in Case 5. Sarcomatous components were observed in the lymphatic invasion site. (**D**, **E**) Hematoxylin–eosin staining (**D**) and vimentin staining (**E**) of the metastatic lymph nodes in Case 5. The tumor cells were positive for vimentin.

In 7 cases (78%), the pathological depth of tumor invasion was shallower than the preoperative clinical diagnosis. Vascular and/or lymphatic invasion was observed in 7 patients, and lymph node metastasis was present in 4. The involved lymph node stations were 105, 106recR, 106recL, 107, 110, 112aoA, 1, 2, 3, and 7, indicating metastases in both mediastinal and abdominal regions. Among these patients, the metastasis consisted of a carcinomatous component (SCC) in 3 cases, while the remaining patient exhibited a sarcomatous component. In all cases, the tumor component (carcinomatous or sarcomatous) at the site of lymphatic invasion was identical to that of the lymph node metastasis (**Fig. 2A**–**2E**).

Five patients experienced recurrence (**Table 2**). Relapse sites included the local site in 2 patients, both in lymph nodes and distant organs in 2 patients, and lymph nodes only in 1 patient. Although treatment strategies for recurrence were tailored to each case, the efficacy of each treatment remained limited. While chemoradiation therapy with cisplatin and 5-fluorouracil achieved a transient partial response, disease progression was observed shortly thereafter. The median survival time after recurrence was 11 months (range: 1–21 months).

**Table 2:** Cases of recurrence

Case no.	Recurrence site	Time since surgery (months)	Pathological diagnosis of recurrence site	Treatment for recurrence	Clinical response	Survival time after recurrence (months)
3	Local site	14	SCC	Surgery	Regrowth	8
4	Lymph node, lung	24	NA	RT	PD	11
5	Lymph node, adrenal gland	4	NA	BSC	—	1
6	Local site	7	SCC	dCRT	SD	15
9	Lymph node	5	NA	dCRT	PR	21

BSC, best supportive care; dCRT, definitive chemoradiotherapy; NA, not available; PD, progressive disease; PR, partial response; RT, radiation therapy; SCC, squamous cell carcinoma; SD, stable disease

**Fig. 3:**
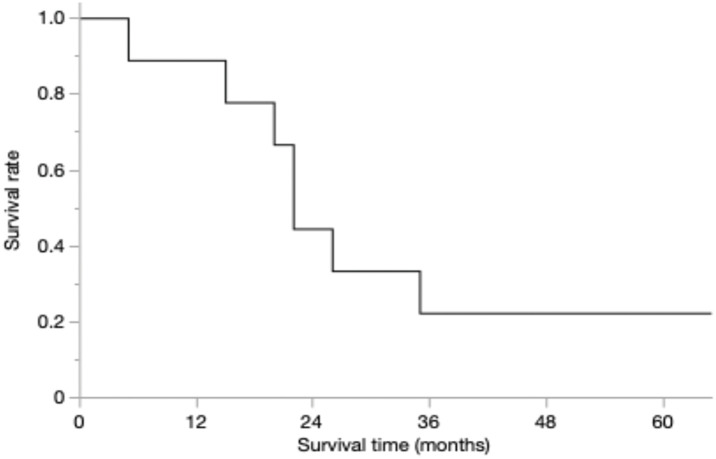
Overall survival for 9 cases of ECS. ECS, esophageal carcinosarcoma

**Fig. 4:**
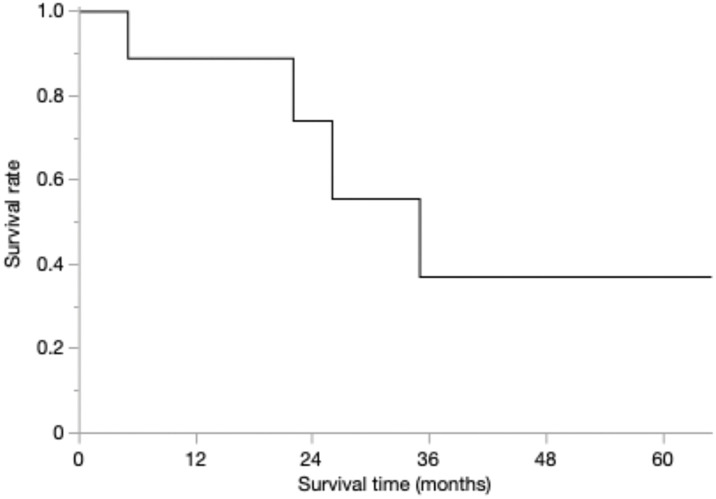
DSS for 9 cases of ECS. DSS, disease-specific survival; ECS, esophageal carcinosarcoma

All patients died during the follow-up period. The causes of death were ECS in 4 cases, other cancers in 2, and other diseases in 3. The median survival was 22 months, and the 5-year overall survival rate was 22.2% (**Fig. 3**). DSS was also analyzed, and the 5-year DSS rate was 37.5% (**Fig. 4**).

## DISCUSSION

In our series, ECS frequently presented as a large polypoid tumor, with a median tumor size of 10 cm, and lymph node metastasis was observed in 4 patients (44%). In addition, accurate preoperative diagnosis of ECS was challenging, with only 1 patient correctly diagnosed by endoscopic biopsy, whereas the majority of cases were initially diagnosed as SCC. These findings were generally consistent with previous reports.^3–11)^ Clinicopathological features, treatment strategies, and outcomes reported in previous case series are summarized in **Table 3**.

**Table 3:** Summary of previous reports of esophageal carcinosarcoma

Author	Year	n	Polypoid-type tumor (%)	Median tumor size (cm)	Lymph node metastasis (%)	Preoperative diagnostic accuracy (%)	Primary treatment	Preoperative therapy	Postoperative therapy	Outcome
Iyomasa et al.^3)^	1990	20	75	7.06	65	NA	Surgery (n = 20)	None	CT (n = 6)	5-year OS: 26.7%
Kuo et al.^8)^	2010	12	66.7	6	50	NA	Surgery (n = 9) CRT (n = 2) CT (n = 1)	CRT (n = 2)	NA	2-year OS: 25% Median OS: 11.5M
Wang et al.^4)^	2013	33	87.8	6	45.5	NA	Surgery (n = 30) CRT (n = 1) Other (n = 2)	None	CT (n = 4) RT (n = 3)	5-year OS: 48% Median OS: 43.5M
Zhang et al.^5)^	2016	71	66.2	5	45.1	14.1	Surgery (n = 70) EMR (n = 1)	None	CT (n =15) RT (n = 13)	5-year OS: 44.8% Median OS: 43M
Katsuya et al.^7)^	2017	19	84.2	6	47.4	NA	Surgery (n = 19)	CT (n = 4) CRT (n = 2)	NA	Median OS: 28.0 months (NAC ± RT) vs. 47.2 months (surgery alone)
Hashimoto et al.^6)^	2019	28	78.6	6.05	50	17.9	Surgery (n = 28)	CT (n = 2)	CT (n = 9) RT (n = 3)	5-year OS: 61.9%
Chen et al.^10)^	2021	24	NA	6	33.3	NA	Surgery (n = 19) CRT (n = 4) RT (n = 1)	CT (n = 1)	CRT (n = 3) CT (n = 3) RT (n = 1)	5-year OS: 70.8%
Shen et al.^11)^	2024	16	62.5	NA	50	31.2	Surgery (n = 16)	CT (n = 2)	CRT (n = 2) CT (n = 4) RT (n =1)	5-year OS: 57.1%
Present study	2026	9	78	10	44	11	Surgery (n = 9)	None	CT (n = 3)	5-year OS: 22.2%, 5-year DSS: 37.5%

CRT, chemoradiotherapy; CT, chemotherapy; DSS, disease-specific survival; EMR, endoscopic mucosal resection; NA, not available; NAC, neoadjuvant therapy; OS, overall survival; RT, radiotherapy

In our series, due to this substantial size, the depth of invasion tended to be overestimated clinically; however, pathological findings revealed relatively shallow invasion in many cases. Despite this, lymphovascular invasion was observed in the majority of patients. These findings suggest that ECS may exhibit aggressive biological behavior that is not adequately reflected by tumor depth alone. Similar clinicopathological findings have been reported previously.^3–11)^ Such discrepancies between invasion depth and lymphovascular invasion have also been reported in superficial esophageal SCC, suggesting that this phenomenon may reflect a biological feature inherent to esophageal cancer rather than being specific to ECS.^12,13)^

**Fig. 5:**
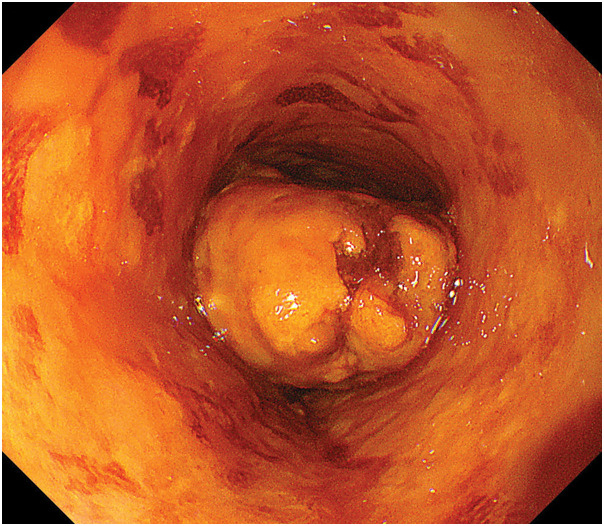
Typical endoscopic appearance of ECS. The polypoid-type tumor presented superficial-type SCC at the base. ECS, esophageal carcinosarcoma; SCC, squamous cell carcinoma

The difficulty in preoperative diagnosis of ECS may be attributed to the biphasic histological nature of ECS. Previous studies have reported low preoperative diagnostic accuracy rates for ECS, ranging from approximately 14% to 31%.^5,6,11)^ ECS is often characterized by a large, protruding, polypoid tumor with superficial SCC at its base (**Fig. 5**).^1)^ Therefore, ECS should be considered in the differential diagnosis when a large polypoid esophageal tumor associated with superficial lesions is encountered. To improve diagnostic accuracy, sampling from at least 2 distinct locations—the protruding tumor component and the tumor base—may be desirable, particularly when ECS is suspected based on macroscopic findings. In addition, a deeper biopsy from the protruding lesion may be helpful to identify the sarcomatous component.^14,15)^

Regarding treatment outcomes, all patients in our series underwent primary surgery, and lymph node dissection was also performed according to the principles used for esophageal SCC. Lymph node metastases were observed in 4 patients, involving both mediastinal and abdominal lymph node stations. There is currently no evidence specifically addressing the optimal extent of lymph node dissection in ECS. However, previous reports have described radical esophagectomy with lymph node dissection as the standard surgical approach for ECS, comparable to that used for esophageal SCC.^3,6,14)^ Considering these findings, reduction of the extent of lymph node dissection may be inappropriate, and at present, an extent of lymphadenectomy similar to that used for esophageal SCC appears to be appropriate.

However, despite these surgical approaches, the prognosis of our patients remained poor. These findings suggest that surgery alone may be insufficient for disease control in some patients with ECS, suggesting the potential importance of multimodal treatment approaches. Furthermore, adjuvant chemotherapy with cisplatin and 5-fluorouracil, as well as chemoradiotherapy for recurrent disease, demonstrated limited efficacy in our cohort. Previous reports similarly suggest that the sarcomatous component of ECS often exhibits low sensitivity to fluoropyrimidine- and platinum-based chemotherapy.^7,14)^ In contrast, several case reports have described responses to taxane-containing regimens in ECS. Specifically, the combination of docetaxel, cisplatin, and 5-fluorouracil has been reported to induce marked regression or even complete disappearance of the sarcomatous component in selected cases, suggesting that taxanes may have specific activity against this component.^16)^ Considering these findings, DCF therapy, a taxane-containing regimen widely used in the neoadjuvant setting for esophageal SCC in Japan, may also represent a potential treatment option for ECS. However, its role in ECS remains to be clarified.

While immune checkpoint inhibitors have gained prominence in treating esophageal cancer, evidence regarding their efficacy in ECS remains extremely limited. To the best of our knowledge, only a single Japanese case report has described a favorable response to nivolumab therapy for postoperative lymph node recurrence of ECS.^17)^ While the efficacy of lenvatinib plus pembrolizumab has been suggested for uterine carcinosarcoma,^18)^ it remains unclear whether immune checkpoint inhibitors can be established as a therapeutic option for ECS. Therefore, further accumulation of clinical experience is warranted.

In addition to these clinical findings, we investigated the pathological characteristics of lymphatic invasion and lymph node metastasis. In our series, the histological component identified within the lymphatic invasion of the primary tumor was consistent with that observed in metastatic lymph nodes. Although the number of cases was limited, these findings suggest a possible association between the histological component at the site of lymphatic invasion and that of lymph node metastases. However, at present, the clinical implications of this finding remain unclear, and it should be considered hypothesis-generating rather than directly applicable to treatment selection. Further studies with larger cohorts are required to validate these findings and clarify their biological and clinical significance.

## CONCLUSION

ECS may exhibit aggressive biological behavior that is not adequately reflected by tumor depth alone, and surgery alone or conventional fluoropyrimidine- and platinum-based chemotherapy may be insufficient for disease control in some patients. Further studies are required to clarify the biological characteristics of ECS and to establish optimal treatment strategies.

## DECLARATIONS

### Availability of data and materials

The authors declare that all data in this article are available within the article.
